# Community-Based Management of Child Malnutrition in Zambia: HIV/AIDS Infection and Other Risk Factors on Child Survival

**DOI:** 10.3390/ijerph13070666

**Published:** 2016-07-01

**Authors:** Stefania Moramarco, Giulia Amerio, Clarice Ciarlantini, Jean Kasengele Chipoma, Matilda Kakungu Simpungwe, Karin Nielsen-Saines, Leonardo Palombi, Ersilia Buonomo

**Affiliations:** 1 Department of Biomedicine and Prevention University of Rome Tor Vergata, via Montpellier, Rome 00133, Italy; stefania.moramarco@gmail.com (S.M.); leonardo.palombi@gmail.com (L.P.); ersilia.buonomo@uniroma2.it (E.B.); 2 Rainbow Project Association Pope John 23rd, 5656 Chinika Road, Ndola 10101, Zambia; giulia.amerio@gmail.com (G.A.); clarice.ciarlantini@gmail.com (C.C.); 3 Ndola District Health Office, 1307 Naidu Close, Ndola 10101, Zambia; jeankasengele@gmail.com (J.K.C.); mkakungusimpungwe@yahoo.co.uk (M.K.S.); 4 Department of Pediatrics, David Geffen UCLA School of Medicine, Los Angeles, CA 90095, USA

**Keywords:** child malnutrition, community-based management of malnutrition, HIV, child survival, supplementary feeding programs, Zambia

## Abstract

(1) Background: Supplementary feeding programs (SFPs) are effective in the community-based treatment of moderate acute malnutrition (MAM) and prevention of severe acute malnutrition (SAM); (2) Methods: A retrospective study was conducted on a sample of 1266 Zambian malnourished children assisted from 2012 to 2014 in the Rainbow Project SFPs. Nutritional status was evaluated according to WHO/Unicef methodology. We performed univariate and multivariate Cox proportional risk regression to identify the main predictors of mortality. In addition, a time-to event analysis was performed to identify predictors of failure and time to cure events; (3) Results: The analysis included 858 malnourished children (19 months ± 9.4; 49.9% males). Program outcomes met international standards with a better performance for MAM compared to SAM. Cox regression identified SAM (3.8; 2.1–6.8), HIV infection (3.1; 1.7–5.5), and WAZ <−3 (3.1; 1.6–5.7) as predictors of death. Time to event showed 80% of children recovered by SAM/MAM at 24 weeks. (4) Conclusions: Preventing deterioration of malnutrition, coupled to early detection of HIV/AIDS with adequate antiretroviral treatment, and extending the duration of feeding supplementation, could be crucial elements for ensuring full recovery and improve child survival in malnourished Zambian children.

## 1. Introduction

Zambia is a sub-Saharan country facing a high burden of child acute malnutrition, with malnutrition remaining one of the most serious problems among children under five years of age [1]. According to the Zambian Preliminary Report of Demographic and Health Survey 2013–2014, 40% of children are affected by stunting, 15% are underweight, and 6% of children suffer from wasting, with a high under-five mortality rate (75 deaths per 1000 live births in a year) [2]. Malnourished children who do not quickly break away from the vicious cycle of infectious disease and growth failure are vulnerable to irreversible cognitive damage [3]. Acute malnutrition (wasting), especially in severe form, if untreated, is an attributable cause of the 12.6% of the 6.9 million deaths worldwide among children under five years of age [4]. In Zambia, malnutrition has been estimated to underlie up to 52% of all under-five deaths [5]. The picture of malnutrition is exacerbated by the HIV/AIDS pandemic: when the condition of being HIV-positive coexists with malnutrition, the risk of growth failure and morbidity increases, and children delay recoveries and suffer relapses of malnutrition events [6,7], with a longer need of nutritional rehabilitation therapy compared to their HIV-negative counterparts [8]. A most recent research demonstrates that acute and chronic malnutrition decrease the odds of attaining adequate motor milestones even in HIV-exposed, but not infected, children [9]. The Zambian Government has been committed to address child malnutrition, by implementing important health sector reforms aimed at strengthening health service delivery in order to improve the health of Zambian children [10]. The process started in 2005, with the community-based therapeutic care (CTC) for the management of malnutrition first introduced in Lusaka district [11]. The community-based management of malnutrition has been endorsed at the international level as an innovative cost effective approach for managing child malnutrition, integrated into the routine health system of many African countries, preventing hundreds of thousands of child deaths when applied at scale [12]. This model of intervention is combining facility- and community-based approaches, which reserve inpatient care (IC) based on the World Health Organization (WHO) recommended management protocol for malnourished children with medical complications [13], and outpatient therapeutic care (OTP) complemented by supplementary feeding programs (SFP) for uncomplicated cases [14]. Targeted supplementary feeding programs (SFPs) are particularly effective in the management of acute malnutrition, treating moderate acute malnutrition (MAM) and preventing the deterioration into severe acute malnutrition (SAM); however, the management of MAM is still debated and consensus regarding management has still not been reached, particularly in non-emergency situations and relatively food-secure settings in low and middle-income countries [15]. SFPs in Zambia are not widely available, and most areas are not covered by nutrition-specific interventions targeting MAM. Rainbow Project SFPs are the only well implemented sites with this specific approach in the Copperbelt. There is a need for more evidence-based data reporting on the effectiveness of SFPs, not only to address areas of weakness and plan new interventions, but also to support the potential need for greater implementation of SFPs in Zambia, taking into consideration different scenarios.

## 2. Materials and Methods

### 2.1. Setting

The current study presents data on malnourished Zambian children assisted in the Rainbow Project SFPs. The Rainbow Project, under the Pope John 23rd Association, is a large-scale model of care for orphans and vulnerable children operating since 1998 in the Ndola and Kitwe Districts. The model is made by several components, including the community-based program against malnutrition. In the context of a traditional CMAM, Rainbow Project is running SFPs with a particular focus on the community mobilization and capacity building activities. In the Ndola District, 11 SFPs are present in different areas around the city. All of the centers are run by leaders of ten small non-governmental organizations (NGOs) and community-based organizations (CBOs), and are coordinated by professionals of Rainbow office, in close network with Ndola District Health Management Teams (DHMTs) and other local authorities. All the SFPs activities are performed by community volunteers/operators appropriately trained in Infant and Young Child Feeding (IYCF) practices promoted by the Zambian Government and who are constantly updated on nutrition topics. Personnel are also trained in confidentiality issues (e.g., counseling in the context of prevention of mother-to-child transmission PMTCT) [16].

### 2.2. Malnutrition Classification, Management and Integration of CMAM Components

Malnourished children enrolled in Rainbow SFPs were recruited through community outreach or referred from local health facilities. Children were admitted to SFPs by using a two-priority criteria system of enrollment: first priority was given to acute malnutrition (SAM or MAM) and second priority to underweight status. If a child qualified at the same time for different criteria, the enrollment was made considering the most severe condition of malnutrition. According to WHO/UNICEF criteria [17] and the Integrating Management of Acute Malnutrition (IMAM) guidelines of the Zambian Ministry of Health [18] for children aged 6 to 59 months, SAM was defined as a weight-for-height (WHZ) or a weight-for-length z-score (WLZ) ≤ −3, mid-upper arm circumference (MUAC) ≤ 11.5 cm, and/or presence of bilateral pitting edema (kwashiorkor); MAM was defined as WHZ/WLZ < −2 and >−3, MUAC ≤ 12.5 cm and >11.5 cm. As recently recommended by the updated WHO guidelines on the management of severe acute malnutrition in infants and children, in order to achieve early community identification of malnourished children, Rainbow’s well-trained volunteers measured the MUAC and examined children for pitting edema, allowing the assessment of WHZ/WLZ to be done by health workers within primary health care facilities and hospitals [19]. According to the WHO standard, underweight was defined as a weight-for-age z-score (WAZ) < −2 [20]. Children identified as having SAM were referred to the Arthur Davidson Children’s Hospital for inpatient treatment if medical complications were present, or to the OTP in the absence of medical complications. All children with MAM were eligible for SFPs: children with MAM without health complications were directly enrolled, while children with MAM with health complications were first to be referred to the nearest health facility for immediate medical care. For ethical and humanitarian reasons, Rainbow SFPs admitted children with SAM or health complications despite additional referral for admission to the nearest health facility for proper screening and advice from the health staff. SFP admission was made possible when CMAM was not fully implemented or access to the hospital was restricted (i.e., OTPs were not covering the whole Ndola area, the availability of ready-to-use therapeutic food/RUTF was erratic, inpatient care was not provided or not applicable), in order to facilitate access to food for more severe cases.

### 2.3. SFP Activities and Data Collection

On a weekly basis, the SFP protocol included anthropometric follow-up, data recording, meals in loco (porridge meal), nutritional supplementation, home visits, as well as health talks and cooking demonstrations for mothers/care-givers. Anthropometric follow up consisted in measuring weight, MUAC, and checking for bilateral pitting edema. Children were assessed without clothes or footwear; weight (in kilograms) was measured using a mechanical baby scale graduated by 0.1 kg increments (salter 235). Mid-upper arm circumference (in centimeters) was measured using a simple colored plastic strip (standardized UNICEF tape). Bilateral pitting edema was checked by applying gentle thumb pressure on the dorsum of the feet and assessing for residual depression; edema was detected as different grades.

Data was collected on general and socio-demographic characteristic, as well as health and nutritional conditions. All information was entered in a register edited ad hoc by the Rainbow Project, in order to monitor health and nutritional status of the beneficiaries. General demographic pediatric information included date of birth, age, gender, and siblings. Socio-demographic data recorded included family history (parents’ marital status, relationship and age of the guardian), and housing information (area of stay, address, household conditions). Health information included disability, medical complications and/or illness, enrollment in OTP, and HIV status. The latter (HIV status), was ascertained from the PMTCT section of the child under-five card released from the primary health care facilities of the Zambia Health Ministry. Nutritional parameters included anthropometric measurement (weight, MUAC, edema). Data was collected after verbal consent of caregivers and in full respect of confidentiality.

Educational activities for mothers/guardians included provision of health talks and cooking demonstrations. A meal in loco was offered to all of the children attending the program. It mainly consisted in porridge, prepared with a high-energy protein supplement (HEPS). The high-energy protein supplement is a specific corn-soya blended food (CSB), fortified with micronutrients (vitamins and minerals), mostly recommended for the management of malnutrition in SFP. Children received on a weekly basis a food ration to take home as nutritional supplementation. Considering that SFPs were conducted in food insecure areas where availability of food was generally limited, local food for the whole household was distributed in addition to HEPS, so that the specific supplement for the child was not shared within the family. Children with SAM without medical complications, if enrolled in the OTP, received RUTF from health facilities, when available.

Home visits were performed by community volunteers to ensure compliance with nutritional and health guidelines. All children stayed in the program until SFP discharge criteria were met: for two consecutive weeks edema should be absent and MUAC > 12.5 cm, or 15% weight gain had to be considered if underweight was the admission criteria.

### 2.4. Study Population, Statistical Analysis, and Ethical Considerations

This was a community-based retrospective observational study. Pediatric data was routinely collected and entered in the database with removal of personal identifiers. The current study presents data on malnourished Zambian children aged 6 to 59 months who were assisted from 2012 to 2014 in the Rainbow Project SFPs around the Ndola area. A database with all pediatric records coming from different SFPs was generated. Data were extracted and analyzed using SPSS software system 20.0 (IBM, Somers, NY, USA). Weight-for-age z-scores (WAZ) were calculated using the WHO Anthro Software (Version 3.2.2, January 2011, WHO, Geneva, Switzerland), based on the 2006 World Health Organization Child Growth Standards [20]. Descriptive data and variables measured were presented as means with standard deviations (SD). For recovered children differences between means of anthropometric parameters were tested with the student *t*-test. The odds ratios, 95% confidence intervals, between age and length of stay, age and SAM, HIV, and MAM were calculated. Univariate and multivariate Cox regression (forward stepwise model) was performed to identify the main predictors of mortality and cure.

The study protocol was approved by the Tropical Diseases Research Centre (TDRC) Ethics Committee of Ndola, Zambia (IRB registration number 00002911).

### 2.5. Program Outcomes and Performance Indicators

In order to evaluate program performance, exit categories for targeted SFPs from Sphere Project and UNHCR guidelines were considered. The Sphere Project defines the standards by which the international community responds to the plight of people affected by disasters, principally through a set of guidelines that are set out in the Humanitarian Charter and Minimum Standards in Humanitarian Response. Sphere standards are the typical criteria used for assessing the effectiveness of SFP [21]. UNHCR guidelines are intended as a practical guide to design, implement, monitor, and evaluate selective feeding programs in emergency situations [22].

Standard outcomes were defined as recovery rate, death rate, and default rate. Recovered/cured was defined as an individual who met the discharge criteria. Defaulter was defined as a child lost to follow up for three consecutive weeks. A child was classified as “defaulter” when he/she dropped out of the study due to refusal or it was not possible to locate the child and make a home assessment. Death was registered when occurring during the time the patient was enrolled in the program. Early mortality (within 15 days from enrollment) was excluded because it might not be directly attributable to the performance of the SFPs. Individuals who did not complete their treatment because they moved to another area were considered transferred. This outcome was not included in the performance evaluation because of the current absence of published targets. Length of stay and weight gain were considered additional indicators for targeted SFPs [23]. Mean length of stay expressed the average time of stay for recovered children; mean weight gain expressed the average number of grams gained per kg per day among children who were cured. For humanitarian and ethical reasons treatment was provided until children reached the recovery goals (treat-to-goal), so none were categorized as non-cured/non-responder (defined as cases that did not reach discharge criteria after a pre-defined length of time). We reviewed the literature in order to compare Rainbow Project’s performance with published outcomes of other similar programs.

## 3. Results

**Figure 1:**
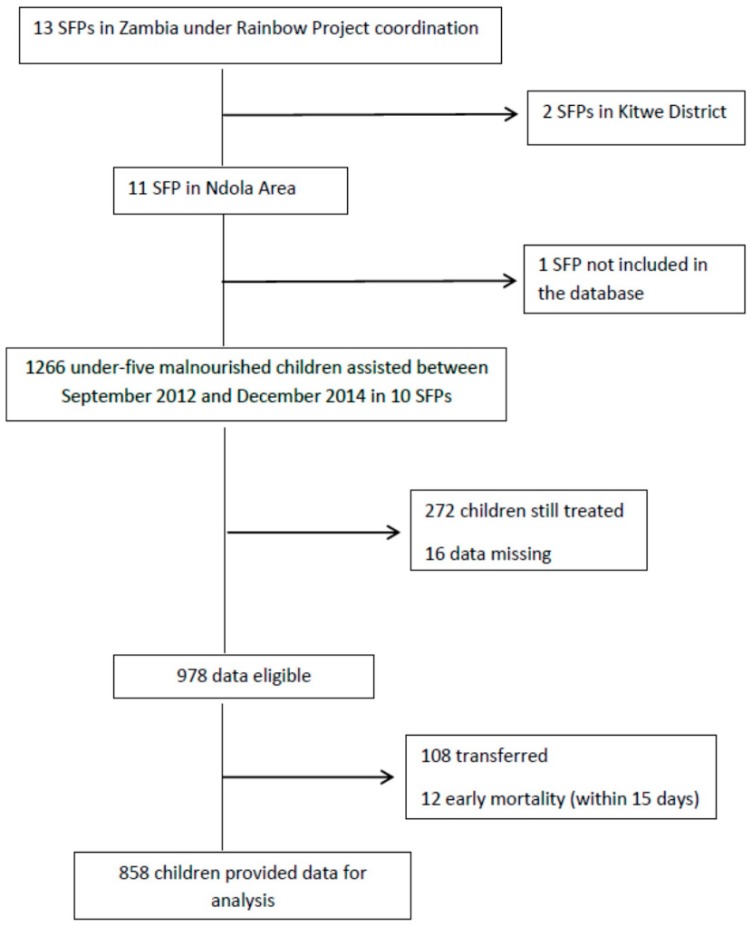
Study flowchart.

Information about SFPs within the Ndola area were considered for this study. Specifically, we focused on 10 SFPs, eight operating in urban areas (Twapia, Nkwazi, Kabushi, Kaloko, Kawama, Chifubu, and Mackenzie), and two located in rural areas (Baluba and Chikumbi); only one center was not entered in the database. Data for 1226 children, all coming from low socio-economic households and assisted from 2012 to 2014, were extracted from the database. Children still on SFPs treatment at the moment of the study were not included. Formally transferred and early mortality episodes were excluded. Twelve cases were left out because of missing data. A total of 858 children (49.9% male) provided data for the analysis (Figure 1).

Table 1 reports the demographic, social, health, and nutritional characteristics of children at baseline. The median age was 19 months ± 9.4 SD, with a range from 5 to 60 months. More than half of the sample was less than 18 months of age (53%). Children younger than 18 months were more likely to be severely malnourished (OR 1.5 CI 1.1–2.1). Regarding the admission criteria, 37.6% of children were affected by MAM, 28.1% were affected by SAM, and 34.3% were admitted due to being underweight. Bilateral pitting edema was identified in 9% of the children. The mean weight of the sample was 7.8 kg ± 1.6 SD; the mean MUAC was 12.4 cm ± 1 SD; the mean WAZ was −2.8 ± 1.1 SD. Sixty-eight children (7.9%) suffered relapses of malnutrition (defined as a new episode of malnutrition after previous discharge). At time of admission, health problems affected 34.9% of beneficiaries; specifically 8.5% had fever, 8.3% had diarrhea, 7.7% had lack of appetite, 3.3% had cough, 0.8% had malaria, and 6.3% had other unspecified conditions. As measures of health problems, we asked the mother/caregiver if the child was experiencing any illness at the moment of the enrollment in the SFP. No physical examinations were performed.

**Figure 2:**
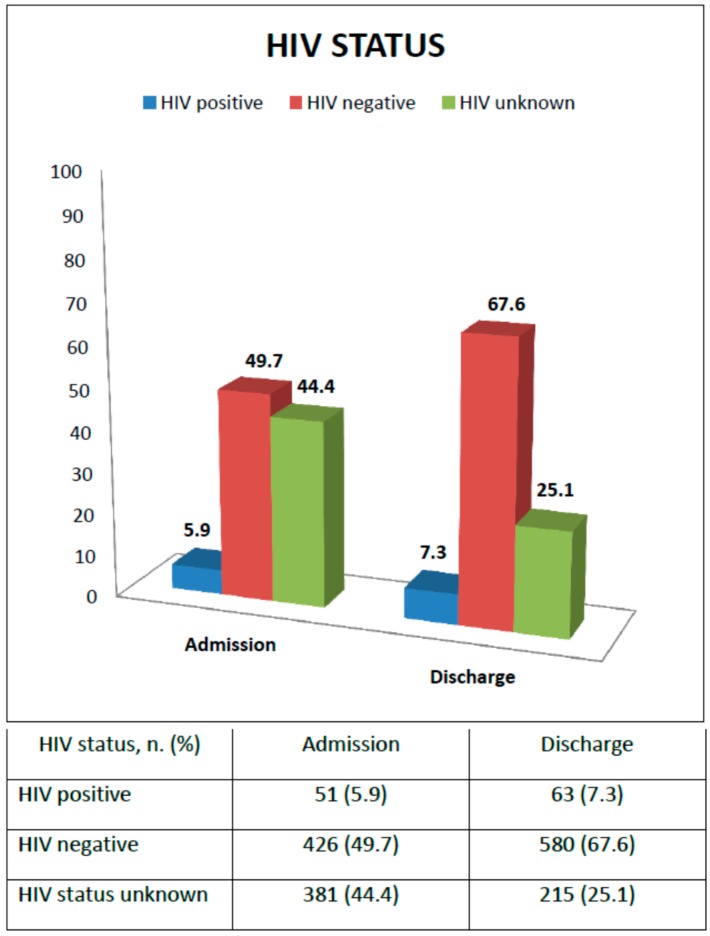
Diagnosis of HIV status from enrollment to discharge.

Figure 2 shows HIV status at admission and discharge. At enrollment, 51 children were reported as HIV infected (5.9%), 426 HIV uninfected (49.7%), while for 381 children the HIV status was unknown (44.4%). Supporting voluntary counseling and testing (VCT) was an SFP activity: all guardians of children with unknown HIV status were encouraged to go to the nearest health facility for an HIV rapid test for both mother and child; HIV-positive children not receiving ARV treatment were referred to the ART clinic for assessment of ART eligibility. At the time of discharge, 63 children (7.3%), that is 12 more children, were found to be HIV infected with 33 (52.3%) initiating ARV treatment, and six (18.2%) also initiating TB treatment; 580 children (67.6%) were confirmed to be HIV-negative, while still 215 children (25.1%) had an unknown HIV status. For only 2.2% HIV exposure was ascertained from the health cards. For the others it was not even possible to know the exposure to HIV.

### 3.1. Performance of the Rainbow Project Based on International Standards

In Table 2 outcomes of the Rainbow Project, reported as for total, MAM, and SAM, were compared with international Sphere standards and UNHCR guidelines for the community-based management of acute malnutrition.

Outcomes for the three main core performance indicators (recovery, death, and defaulter rate) were compared with Sphere standards. The overall recovery rate was 82.6%; respectively, 86.1% for MAM and 73.5% for SAM. The global default rate was 11.8%, with 11.1% MAM and for 14.1% for SAM, all below the standard. The overall case-fatality rate was 5.6%, above the Sphere standard. When splitting the two groups, the death rate for MAM was below the target (2.8%), while the case-fatality rate for SAM was 12.4%. The number of deaths, which is above the alarming target was mainly due to the very critical health and poor nutrition conditions of these children associated with the low access to adequate health care. Two other targeted SFP indicators, length of stay and weight gain, were considered. The mean length of stay was 19.3 weeks; compared to International Standards, an increased number of weeks was needed for recovery of children with MAM (+7 weeks), and an even longer period for children with SAM (+10 weeks). Children younger than 18 months of age were more likely to stay longer (OR 1.4 95% CI: 1.1–1.8). The average weight gain was generally 1.7 g/kg/day. The mean weight gain for MAM was 1.7 g/kg/day, below the international guidelines (UNHCR). Published data reports a desirable weight gain of ≥5 g/kg/day [24], while it tended to be less in some studies (3 g/kg/day) [25] and reviews of the literature (between 1 and 2 g/kg/day) [26]. The mean weight gain of severely-malnourished children (2 g/kg/day) was far below that of UNHCR guidelines and recent literature (4.4 g/kg/day) [27].

### 3.2. Anthropometric Analysis of Recovered Children

In order to assess the nutritional status of recovered children, differences between anthropometric parameters’ means at admission and discharge (weight, WAZ, MUAC) were compared using Student’s *t*-test. Table 3 shows significant improvements (*p* < 0.0001) on the anthropometric parameters: the weight gained from 7.8 kg ± 1.6 to 9.2 kg ± 1.6 (+1.4 kg ± 0.8); the WAZ rose from −2.8 ± 1.1 to −1.9 ± 0.9 (+0.8 ± 0.8); and the MUAC increased from 12.4 cm ± 1 to 13.7 cm ± 0.8 (+1.3 cm ± 0.9).

### 3.3. Predictors of Mortality and Cured

We performed a univariate and multivariate (forward stepwise model) Cox proportional risk regression to identify the main predictor of mortality (Table 4). Variables of severity of malnutrition, HIV status, weight gain, weight for age z-score at admission, age in months and frequency of health problems, and health problems at admission were included in the analysis. Both analyses showed a significant association with SAM, HIV infection, and WAZ < −3 at admission.

**Figure 3:**
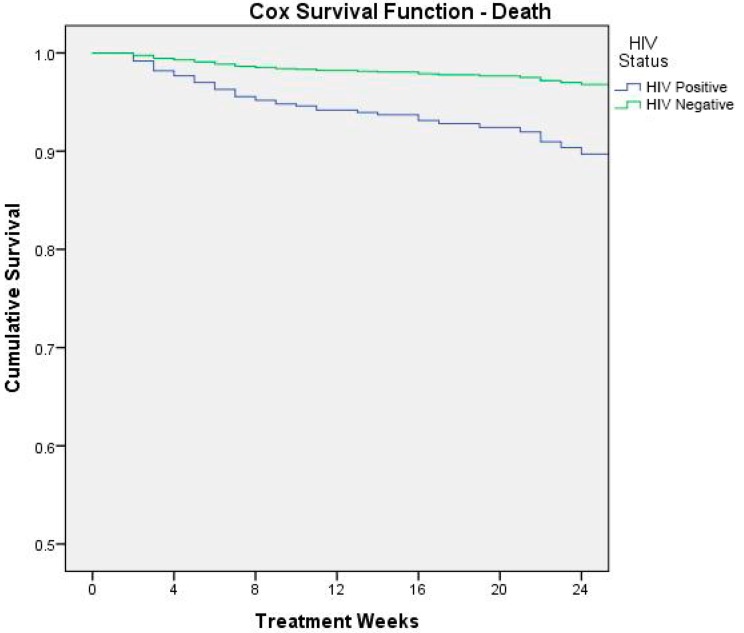
Cox survival analysis (outcome: death) by HIV status.

Figure 3 shows the result of Cox survival analysis (outcome: death) by HIV status.

Finally, we performed a Cox analysis using as the dependent variable the “cured” status, calculating the time to event as seen in Table 5.

**Figure 4:**
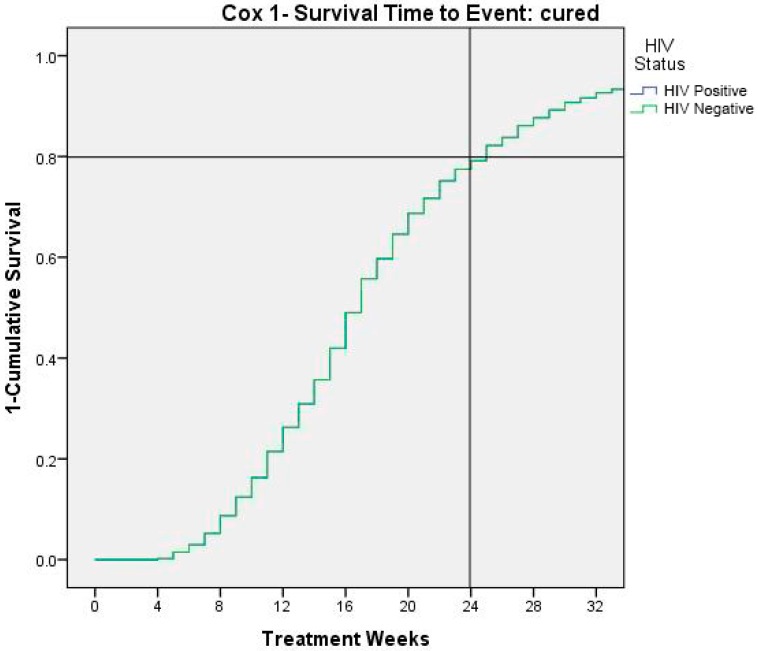
Time to cure events as 1- survival.

Figure 4 shows time to event (cured children). At 24 weeks 80% of children result recovered by severe/moderate malnutrition.

## 4. Discussion

The main objective of this study was to evaluate the effectiveness of the model in the community management of child malnutrition in the Ndola district. Rainbow Project SFPs in the Ndola area assisted children from 6 to 59 months old with MAM and, for humanitarian and ethical reasons, children with SAM and/or health complications when CMAM was not fully implemented. Rainbow SFP activities included both nutrition-specific or direct interventions (growth monitoring and supplementary food) and nutrition-sensitive or indirect interventions (HIV counseling and testing, nutrition, and health skills for mother and child health promotion). General outcomes showed good program performance. Outcomes for MAM and SAM were analyzed separately in order to better compare the program performance with International Standards. Results of this study demonstrate that Rainbow SFPs is very effective in the management of MAM. Outcomes for MAM met all the Sphere standards for targeted SFPs, with high recovery rates (86.1%), and both low defaulter (11.1%) and mortality rates (2.8%). Comparing with other studies, our findings showed outcomes similar to other community-based programs for moderate malnutrition, or in some cases with a better cure rate but higher mortality rates [25,27,28]. Outcomes for children with SAM exceeded the international standards. Our findings reflected the most critical condition of severely malnourished children, for whom therapeutic care must be provided, especially when health complications coexisted. These findings were in accordance with a study on community-therapeutic care in Lusaka reporting a mortality for children having SAM treated with RUTF of more than 9%, while in the absence of treatment the mortality rate was expected to be above the 20% [11].

In order to better understand the reasons linked to the fatality rate, we identified a higher risk of mortality, respectively, for severe acute malnutrition, HIV infection, very low weight-for-age z-score at admission, and health problems at admission. These variables were all independent and strong predictors of mortality. With respect to HIV status, our analysis reported that HIV-infected children were almost three times more at risk of death than HIV uninfected. These results, according to Munthali and colleagues, demonstrate that Zambian malnourished children who are HIV-infected are 80% more likely to die than those who are HIV-uninfected [29], highlighting the role of the vicious cycle between HIV/AIDS infection and childhood malnutrition and its negative synergic impact on mortality. In setting where HIV infection is common (in Zambia 100,000 children aged 0 to 14 years are living with HIV [30]), the management of HIV/AIDS malnourished children remains more critical, since nutritional rehabilitation must be essentially integrated with early diagnosis efforts and the adequate ARV therapy [31]. At the moment of data collection the country had adopted WHO Option A as the national ART program for HIV/AIDS treatment and prevention. HIV-positive children below 24 months were recommended to initiate ART irrespective of CD4 counts or WHO HIV clinical stage; children between 24 and 59 months were recommended to initiate ART with CD4 counts of ≤750 cells/mm^3^ or % CD4+ <25%, irrespective of WHO HIV clinical stage, or with WHO HIV clinical stages 3 and 4, irrespective of CD4 counts. Zambian guidelines for option B+ were launched in 2013 and progressively implemented. These new guidelines recommend lifelong triple-combination ART for all confirmed HIV-infected children regardless of CD4 count and/or WHO clinical stage.

Finally, the Cox analysis showed that recovering from severe-moderate malnutrition was reached at 80% of the population only at the end of month six. International guidelines recommend that the length of treatment of 12 weeks but our study shows that further benefits could be added by a prolonged treatment until week 24.

Despite the incisive work Rainbow SFPs performed in HIV counseling and testing, our analysis highlight high percentage of children who remained with an unknown HIV status at time of ending the program, underscoring that stigma HIV and fear of discrimination still bear in Zambia. We can presumably suppose that some of the children with HIV status unknown at the time of discharge from SFP could be HIV infected and, therefore, at more risk of morbidity and mortality. National guidelines must, therefore, be efficiently implemented and PMTCT programs must be improved, both at the community and at the health system level. Enhancing the integration among the different stakeholders dealing at local level with HIV and malnutrition could facilitate early detection of HIV infected children at most need of health care and support. Further research will also need to evaluate the positive health impact of both children and their family since shifting to the WHO 2013 guidelines in Zambia [32].

The low average weight gain suggests that the ration of high-quality food supplementation could be improved. A new food distribution consisting of redoubling the amount of HEPS could be suggested, in order to provide a daily ration of 150 g of HEPS per child. The ration of general local food would be still provided in order to support the household’s diet, considering the poor setting and food sharing within the nuclear family. Assuming that, to meet their daily dietary needs, children rely only on food supplements distributed by such programs, we estimate children could benefit from more than 1000 kcal/day, from either HEPS and local food, with a mean average of more than 25 g/day of protein. This new food schedule could meet the proposed recommended nutrient requirements for moderate malnourished [24], enhancing the effectiveness of the program. Although cost assessment was beyond the scope of this study, we have estimated the new food schedule to be sustainable, not affecting previous monthly costs for SFPs. Presumably, with a better weight gain due to the new food schedule, the mean length of stay for recovered children could decrease, improving the cost-effectiveness of the program in the long-term.

## 5. Conclusions

The Rainbow Project SFPs are effective and sustainable in the community management of child malnutrition in the Ndola district. Results from the Rainbow Project SFPs suggest that preventing deterioration in severe acute malnutrition, coupled to early detection of HIV/AIDS with adequate antiretroviral treatment, and extending the duration of feeding supplementation, are associated with an effective weight gain. These are crucial elements for ensuring full recovery and lowering mortality rates in malnourished Zambian children. In order to improve the Rainbow Project SFPs performance, a higher-quality food distribution was recently implemented. Meanwhile, enhanced education of community volunteers/operators on child malnutrition and HIV/AIDS knowledge has been reinforced.

## Figures and Tables

**Table 1:** Demographic, social, health, and nutritional characteristics of children at baseline.

Variables (*n* = 858)	Value
Male, n. (%)	428 (49.9)
Age in months, mean (SD)	19 ± 9.4
<18 months of age	455 (53)
>18 months of age	403 (47)
Urban area, n. (%)	695 (81)
Rural area, n. (%)	163 (19)
Orphans, n. (%)	48 (5.6)
Orphans of both parents, n. (%)	15 (1.7)
Disability, n. (%) *	8 (6)
Twins, n. (%)	62 (7.2)
Caregiver’s age, mean (SD)	29 ± 10
(min–max)	(13–78)
Referred from, n. (%)	
Hospital	46 (5.4)
Local health facility	279 (32.5)
Community	533 (62.1)
HIV status, n. (%)	
Infected	51 (5.9)
Uninfected	426 (49.7)
Status unknown	381 (44.4)
Admission criteria: MAM, n. (%)	323 (37.6)
<18 months of age	195 (60.4)
>18 months of age	128 (39.6)
Admission criteria: SAM, n. (%)	241 (28.1)
<18 months of age	146 (60.6)
>18 months of age	95 (39.4)
Admission criteria: Underweight, n. (%)	294 (34.3)
<18 months of age	114 (38.8)
>18 months of age	180 (61.2)
Presence of edema, n. (%)	77 (9)
Relapses of malnutrition event, n. (%)	68 (7.9)
Weight (kg), mean (SD)	7.8 ± 1.6
WAZ, mean (SD)	−2.8 ± 1.1
MUAC (cm), mean (SD)	12.4 ± 1
Health problems, n. (%)	299 (34.9)
Fever	73 (8.5)
Diarrhea	71 (8.3)
Lack of appetite	66 (7.7)
Cough	28 (3.3)
Malaria	7 (0.8)
Others	54 (6.3)

* data available since May 2014.

**Table 2:** Rainbow Project SFPs outcomes reported as for total, MAM and SAM, and compared with International Standards.

Indicators	Rainbow Project SFPs Outcomes			International Standards	
	Total (*n* = 858)	MAM (*n* = 323)	SAM (*n* = 241)	Acceptable	Alarming
**Recovered (%)**	709 (82.6)	278 (86.1)	177 (73.5)	>75% *	<50% *
**Defaulters (%)**	101 (11.8)	36 (11.1)	34 (14.1)	<15% *	>30% *
**Deaths (%)**	48 (5.6)	9 (2.8)	30 (12.4)	<3% for SFPs * <10% for TFPs *	>10% *
**Mean length of stay, weeks (SD)**	19.3 ± 11.5	19.3 ± 11.9	22 ± 11.8	<12 weeks for SFPs (depending on the national guidelines) ^§^ <3–4 weeks for TFPs (IC till full recovery) ^§^ <60 days for TFPs (IC and OTP combined) ^§^	
**Average weight gain, g/kg/day (SD)**	1.7 ± 1.2	1.7 ± 1	2 ± 1.3	≥3 g/kg/day for SFPs ^§^ ≥8 g/kg/day for TFPs (IC till full recovery) ^§^ ≥4 g/kg/day for TFPs (IC and OTP combined) ^§^	

* Sphere Project [20]; ^§^ UNHCR [21]; TFPs = Therapeutic feeding programs.

**Table 3:** Differences between means of the anthropometric parameters at admission and discharge in recovered children (*n* = 709), and Student’s *t*-test.

	Admission	Discharge	Differences between Means	95% Confidence Intervals (CI)		Student *t*-Test	*p*-Value
				Lower	Upper		
**Mean weight (kg), (SD)**	7.8 ± 1.6	9.2 ± 1.6	−1.4 ± 0.8	1.37822	1.50302	45.327	0.000
**Mean WAZ, (SD)**	−2.8 ± 1.1	−1.9 ± 0.9	−0.8 ± 0.8	0.714137	0.832647	25.625	0.000
**Mean MUAC (cm), (SD)**	12.4 ± 1	13.7 ± 0.8	−1.3 ± 0.9	1.2356	1.3689	38.351	0.000

**Table 4:** Predictors of mortality. Cox proportional risk analysis.

Univariate Analysis				Multivariate Analysis		
Predictors of Mortality	HR	95% CI	*p*	HR Exp (B)	95% CI	*p*
**SAM**	3.8	2.1–6.8	<0.001	4.3	2.3–7.9	<0.001
**HIV infection**	3.1	1.7–5.5	<0.001	3.3	1.8–5.9	<0.001
**Low weight gain**	2.1	0.9–5.1	NS (0.08)	3.1	1.3–7.5	0.009
**WAZ < −3 at admission**	3.1	1.6–5.7	<0.001			NS
**Age < 18 months**	1.0	0.5–1.7	NS (0.9)			NS
**Frequency of health problems**	0.9	0.5–1.7	NS (0.9)			NS
**Health problems at admission**	1.7	0.9–3.1	NS (0.07)			NS

**Table 5:** Predictors of failed cured status. Cox proportional risk analysis.

Univariate Analysis				Multivariate Analysis		
Predictors of Failure	HR	95% CI	*p*	HR Exp (B)	95% CI	*p*
**SAM**	1.7	1.1−2.4	0.001	1.8	1.2−2.5	0.001
**HIV infection**	2.2	1.6−3.1	<0.001	2.2	1.6−3.1	<0.001
**Low weight gain**	1.6	1.0−2.5	0.041	1.9	1.2−3.0	0.006
**WAZ < −3 at admission**	0.9	0.6−1.4	NS (0.6)			NS
**Age < 18 months**	0.8	0.5−1.0	NS (0.1)			NS
**Frequency of health problems**	0.8	0.5−1.1	NS (0.1)			NS
**Health problems at admission**	1.1	0.7−1.6	NS (0.06)			NS

## Abbreviations

The following abbreviations are used in this manuscript:

SFPs	Supplementary Feeding Programs
MAM	Moderate Acute Malnutrition
SAM	Severe Acute Malnutrition
OR	Odds Ratio
CI	Confidence Interval
HR	Hazard Ratio
SD	Standards Deviation
MUAC	Mid-Upper Arm Circumference
WAZ	Weight-for-Age Z-score
HIV	Human Immunodeficiency Virus
AIDS	Acquired Immune Deficiency Syndrome
CTC	Community-based Therapeutic Care
IC	Inpatient Care
WHO	World Health Organization
OTP	Outpatient Therapeutic Care
CMAM	Community-Management of Acute Malnutrition
NGOs	Non-Governmental Organizations
CBO	community-based organizations
DHMTs	District Health Management Teams
IYCF	Infant and Young Child Feeding
PMTCT	Prevention Mother-to-Child Transmission
UNICEF	United Nations International Children’s Emergency Fund
IMAM	Integrated Management of Acute Malnutrition
WHZ	Weight-for-Height Z-score
WLZ	Weight-for-Length Z-score
RUTF	Ready-to-use Therapeutic Food
ID number	Identification number
HEPS	High energy protein supplement
CSB	Corn soya blended food
SPSS	Statistical packages for social sciences
TDRC	Tropical Diseases Research Centre
UNHCR	United Nations High Commissioner for Refugees
VCT	Voluntary counseling and testing
ARV	Antiretroviral
ART	Antiretroviral Therapy
TB	Tuberculosis
